# The mitochondrial genome of Dendrobaena tellermanica Perel, 1966 (Annelida: Lumbricidae) and its phylogenetic position

**DOI:** 10.18699/VJGB-23-20

**Published:** 2023-04

**Authors:** S.V. Shekhovtsov, G.V. Vasiliev, R. Latif, T.V. Poluboyarova, S.E. Peltek, I.B. Rapoport

**Affiliations:** Institute of Cytology and Genetics of the Siberian Branch of the Russian Academy of Sciences, Novosibirsk, Russia Institute of Biological Problems of the North of the Far Eastern Branch of the Russian Academy of Sciences, Magadan, Russia; Institute of Cytology and Genetics of the Siberian Branch of the Russian Academy of Sciences, Novosibirsk, Russia; Semnan University, Semnan, Iran; Institute of Cytology and Genetics of the Siberian Branch of the Russian Academy of Sciences, Novosibirsk, Russia; Institute of Cytology and Genetics of the Siberian Branch of the Russian Academy of Sciences, Novosibirsk, Russia; Tembotov Institute of Ecology of Mountain Territories of Russian Academy of Sciences, Nalchik, Russia

**Keywords:** earthworms, Lumbricidae, Dendrobaena tellermanica, mitochondrial genomes, дождевые черви, Lumbricidae, Dendrobaena tellermanica, митохондриальные геномы

## Abstract

Earthworms are an important ecological group that has a significant impact on soil fauna as well as plant communities. Despite their importance, genetic diversity and phylogeny of earthworms are still insufficiently studied. Most studies on earthworm genetic diversity are currently based on a few mitochondrial and nuclear genes. Mitochondrial genomes are becoming a promising target for phylogeny reconstruction in earthworms. However, most studies on earthworm mitochondrial genomes were made on West European and East Asian species, with much less sampling from other regions. In this study, we performed sequencing, assembly, and analysis of the mitochondrial genome of Dendrobaena tellermanica Perel, 1966 from the Northern Caucasus. This species was earlier included into D. schmidti (Michaelsen, 1907), a polytypic species with many subspecies. The genome was assembled as a single contig 15,298 bp long which contained a typical gene set: 13 protein-coding genes (three subunits of cytochrome c oxidase, seven subunits of NADH dehydrogenase, two subunits of ATP synthetase, and cytochrome b), 12S and 16S ribosomal RNA genes, and 22 tRNA genes. All genes were located on one DNA strand. The assembled part of the control region, located between the tRNA-Arg and tRNA-His genes, was 727 bp long. The control region contained multiple hairpins, as well as tandem repeats of the AACGCTT monomer. Phylogenetic analysis based on the complete mitochondrial genomes indicated that the genus Dendrobaena occupied the basal position within Lumbricidae. D. tellermanica was a rather distant relative of the cosmopolitan D. octaedra, suggesting high genetic diversity in this genus. D. schmidti turned out to be paraphyletic with respect to D. tellermanica. Since D. schmidti is known to contain very high genetic diversity, these results may indicate that it may be split into several species.

## Abstract (ru)

Дождевые черви – важная экологическая группа, которая оказывает значительное влияние как на состав почвенной фауны, так и на растительность. Генетическое разнообразие и филогения дождевых червей при этом остаются относительно слабо изученными. В настоящее время большинство работ по генетическому разнообразию дождевых червей основывается на единичных митохондриальных и ядерных генах. В связи с этим для реконструкции филогенетических отношений у дождевых червей перспективными становятся митохондриальные геномы. Почти все работы по этой теме посвящены видам из Западной Европы или Восточной Азии, другие регионы практически не затронуты. В настоящей работе мы провели секвенирование, сборку и анализ митохондриального генома Dendrobaena tellermanica Perel, 1966. Этот вид ранее входил в состав кавказского вида D. schmidti (Michaelsen, 1907) – политипического вида, в пределах которого выделяли множество таксонов. Геном был собран в виде одного контига длиной 15 298 п. н., содержащего типичный набор генов: 13 белок-кодирующих генов (три субъединицы цитохромоксидазы, семь субъединиц NADH дегидрогеназы, две субъединицы АТФ синтетазы, цитохром b), гены 12S и 16S рибосомальной РНК и 22 гена тРНК. Все гены были расположены на одной цепи ДНК. На контрольный регион, находящийся между генами tRNA-Arg и tRNA-His, приходилось 727 п. н. Контрольный регион содержал множество шпилек, а также тандемные повторы мономера AACGCTT. Филогене- тический анализ на основе полных митохондриальных геномов показал, что род Dendrobaena является базаль- ным в семействе Lumbricidae. D. tellermanica оказалась довольно далеким родственником космополитного вида D. octaedra, что говорит о высоком генетическом разнообразии в этом роде. Вид D. schmidti был парафилетичным по отношению к D. tellermanica. Поскольку для D. schmidti характерна очень высокая генетическая изменчивость, можно рассматривать эти данные как свидетельство в пользу разделения D. schmidti на несколько видов.

## Introduction

Earthworms are an important ecological group that accounts for the highest biomass among the soil fauna in many habitats (Hendrix et al., 2008). Its representatives process plant detritus to soil humus and return organic matter to the global cycles (Blouin et al., 2013). Earthworms also form soil structure, which has high impact on both soil fauna composition and vegetation (Lavelle et al., 2016). Therefore, this group defines ecosystem productivity in many respects.

Genetic diversity and phylogeny of earthworms remain insufficiently studied (Marchán et al., 2018). Currently, most works on earthworm genetic diversity are based on single mitochondrial and nuclear genes (Jamieson et al., 2002; Marchán et al., 2022). Construction of multigene nuclear datasets is impeded by frequent polyploidy characteristic for this group (Viktorov, 1997; Vsevolodova-Perel, Bulatova, 2008; Mezhzherin et al., 2018), which makes it hard to detect suitable orthologs and amplify them by PCR.

Mitochondrial genomes are thus a promising tool for reconstruction of phylogenetic relationships in earthworms. A lot of mitochondrial genomes were sequenced and published in recent years (Zhang L. et al., 2014–2016a, b; Wang et al., 2015; Conrado et al., 2017; Hong et al., 2017; Shekhovtsov, Peltek, 2019; Zhang Q. et al., 2019; Liu et al., 2020; Seto et al., 2021; Csuzdi et al., 2022; Kim, Hong, 2022), and studies on phylogenetic relationships of certain groups were also conducted (Shekhovtsov et al., 2020a; Liu et al., 2021). However, almost all of these studies were made on species from West Europe and East Asia, with almost no representatives of other regions.

In this study, we performed sequencing, assembly, and analysis of the mitochondrial genome of Dendrobaena tellermanica Perel, 1966. This species was earlier included into D. schmidti (Michaelsen, 1907), a polytypic species that was considered to contain multiple subspecies (Michaelsen, 1907; Kvavadze, 1985). D. tellermanica was believed to be a parthenogenetic form of D. schmidti (Perel, 1966). T.S. Vsevolodova- Perel (2003) demonstrated that many populations of D. tellermanica are amphimictic (sexual) and so isolated it into a separate species. D. tellermanica differs from D. schmidti by the lack of pigmentation, different position of the clitellum and the form of tuberculae pubertatis (Vsevolodova-Perel, 2003).

Currently, there is only one complete mitochondrial genome of the genus Dendrobaena in GenBank belonging to the cosmopolitan D. octaedra (Savigny, 1826). The mitochondrial genome of D. tellermanica will be the first sequenced mitochondrial genome of a Caucasian earthworm and will be important for studying the phylogeny of lumbricids

## Materials and methods

Specimens of D. tellermanica were collected in the Karachay- Cherkess Republic (right bank of r. Uchkulan, road to the Chiper pass, 1483 a. s. l., 4–5 km from the Aktyube town, Alchemilla and Geranium meadow, N 43.410944, E 42.174538). Worms were fixed in ethanol. Morphological identification was performed according to the key of T.S. Vsevolodova- Perel (1997).

DNA was extracted using the standard phenol-chloroform method and sonicated on Covaris M220 to the target fragment length of 350 bp. The fragments were purified by 1.2 volume of AMPureXP (Beckman Coulter, USA) and quantified using fluorometry on a Qubit device. Genomic libraries were obtained from 100 ng of DNA using Roche KAPA Hyper Prep according to the manufacturer’s protocol using KAPA UDI Adapter double barcodes. Quality and molarity of the obtained genomic library was assessed on a BA2100 bioanalyzer using the Agilent DNA High Sensitivity Kit and sequenced on an Illumina NextSeq550 with the Mid Output Kit v. 2.5 (300 Cycles) for 2×150 bp paired reads

The obtained data were processed by TrimmomaticPE (Bolger et al., 2014) with the ILLUMINACLIP:TruSeq3- SE:2:30:10 SLIDINGWINDOW:4:15 MINLEN:36 options. SPAdes v. 3.14.1 was used for contig assembly (Bankevich et al., 2012) with the --isolate option. The assembled contigs were aligned with mitochondrial earthworm genomes from the NCBI database with blastn (https://blast.ncbi.nlm.nih.gov) in order to search for mitochondrial sequences.

Preliminary annotation was done by MITOS 2 (Bernt et al., 2013) with subsequent manual comparison with annotated earthworm genomes. The mitochondrial genome of D. tellermanica was deposited in GenBank under accession number ON960857. Map of the genome was constructed using Benchling (https://www.benchling.com/).

Secondary structures of tRNAs were visualized using MITOS 2 (Bernt et al., 2013); of the control region, using RNAfold Web Server (http://rna.tbi.univie.ac.at/cgi-bin/ RNAWebSuite/RNAfold.cgi) and forna (http://rna.tbi.univie. ac.at/forna/forna.html) (Gendron et al., 2001). Search for tandem repeats was done by Tandem Repeats Finder (Benson, 1999). For phylogenetic reconstructions, mitochondrial genomes were aligned with Clustal Omega (https://www.ebi. ac.uk/Tools/msa/clustalo/); control regions were not included into the alignments. Ambiguously aligned regions were removed with gblocks 0.91b (Castresana, 2000). Earthworm mitochondrial genomes and sequences of the COX1 gene of representatives of the Dendrobaena genus were extracted from GenBank. Phylogenetic trees were built using the Maximum Likelihood approach in RAxML v. 8.2.12 (Stamatakis 2014) with the GTRCAT substitution model; 1000 bootstrap replicates were performed.

## Results

We obtained 4.2 million paired reads for the D. tellermanica genomic library; 3.4 mil- lion remained after processing. Median coverage of genome contigs longer than 500 bp was estimated at 6 (average – 20), median coverage for the mitochondrial contig was 20 (average – 30).

**Fig. 1.**
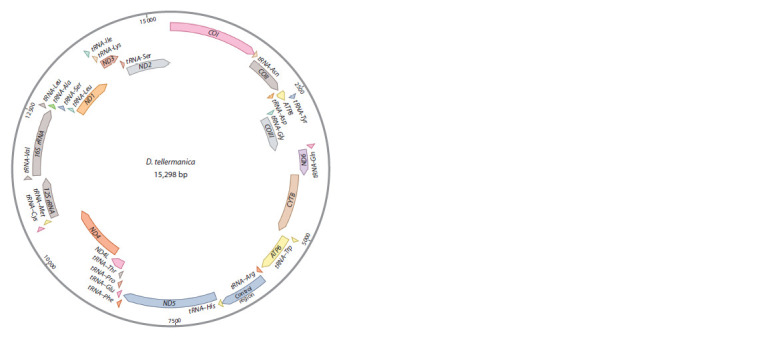
The organization of the D. tellermanica mitochondrial genome.

**Fig. 2.**
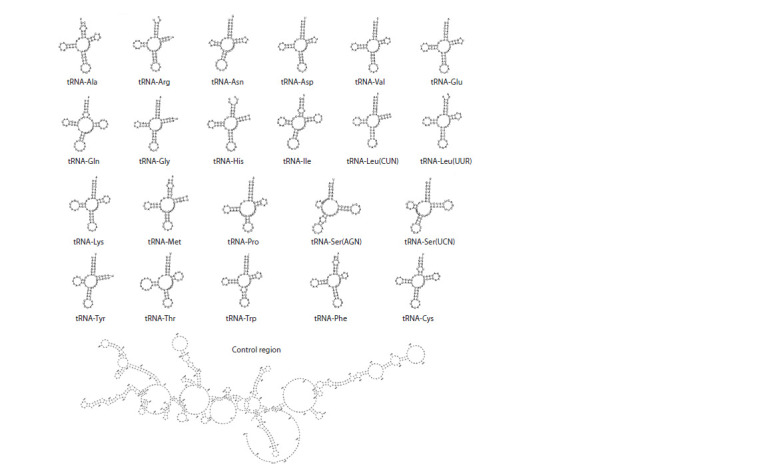
Secondary structures of tRNAs and the control region of the mitochondrial genome of D. tellermanica.

The assembled mitochondrial contig was 15,298 bp long and contained the typical set of genes: 13 protein-coding genes (three subunits of cytochrome oxidase, seven subunits of NADH dehydrogenase, two subunits of ATP synthase, and cytochrome b), 12S and 16S ribosomal RNA genes, and 22 tRNA genes. All genes were located on one DNA strand (Fig. 1). AT- content was 65.3 %. The leading strand contained 31.1 % А, 34.2 % Т, 13.9 % G, and 20.8 % C. The ND4 and ND4L genes overlapped by 7 bp. ATG was the only start codon used. Three protein coding genes (COIII, ND1 и ND2) had an abbreviated stop codon. Transport RNA genes were 60 to 69 bp long, their predicted structures are shown on Fig. 2.

The region between the tRNA-Arg and tRNA-His genes is known as the control region. A total of 727 bp were assigned to it. The control region could not be assembled, so the final sequence contained a gap. Its АТ-content (63.5 %) was close to the genome average, and its sequence contained multiple hairpins (see Fig. 2). It also included 11 tandem repeats of the AACGCTT monomer.

## Discussion

Organization of mitochondrial genome in earthworms

For a long time, the study of J.L. Boore and W.M. Brown (1995) on Lumbricus terrestris was the only description of an earthworm mitochondrial genome. It was 14,998 bp long and contained a set of genes usual for animal mitochondria. The hallmark of earthworm mitochondrial genomes, as well of Annelida as a whole, with few exceptions, is that all genes are located on a single strand (Weigert et al., 2016). All mitochondrial genes in earthworms are presumably expressed as a single transcript (Vallès, Boore, 2006). In this case, any inversions and most of the translocations will be non-viable, which leads to fixed gene positions in the genome. Indeed, mitochondrial gene order in annelids is highly conserved, and all Clitellata have identical gene order. We also failed to find any deviations from this rule.

While mtDNA gene order in earthworms is conserved, its sequence is highly variable, which is especially pronounced for the control region (also referred to as the D-loop). The control region acts as the replication origin, promotor, and the regulatory region for the mitochondrial gene expression (Clayton, 1992).

Organization of the control region varies in different earthworm taxa. Among the representatives of Glossoscolecidae and Megascolecidae they are short, usually less than 500 bp (Zhang L. et al., 2016a; Hong et al., 2017; Zhang Q. et al., 2019; Seto et al., 2021; Kim, Hong, 2022), while in two species of Drawida (Moniligastridae) these sequences were completely absent (Liu et al., 2020). In Lumbricidae, the length of the control region varies from 400 bp in L. terrestris to 2000 bp in Eisenia fetida. For many species, control regions could not be amplified (Shekhovtsov et al., 2020a) or even recovered using NGS methods (Zhao et al., 2022). Here, we also failed to amplify the lacking part of the control region of D. tellermanica. This could be caused by its length or complex secondary structure: Fig. 2 demonstrates that almost all of the control region forms hairpins.

The phylogeny of earthworms based on mitochondrial genomes and the position of D. tellermanica

**Fig. 3.**
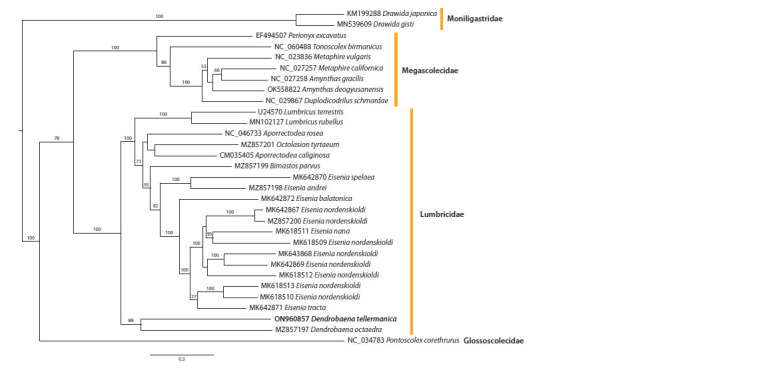
Phylogenetic tree based on earthworm mitochondrial genomes using the Maximum Likelihood method. Here and in Fig. 4: Numbers near the branches indicate bootstrap support.

Phylogenetic analysis based on complete mitochondrial genomes (Fig. 3) suggests that Moniligastridae is distantly related to other earthworm families. Glossoscolecidae, represented here by a single species Pontoscolex corethrurus, occupied the basal position within the order Crassiclitellata. Megascolecidae and Lumbricidae, which were the most densely sampled, turned out as sister groups.

Within the family Lumbricidae, which includes D. tellermanica, the genus Dendrobaena was the sister group to all other genera of the family. There are only two known mitochondrial sequences for the genus Dendrobaena, the cosmopolitan D. octaedra and D. tellermanica obtained in this study. Expectedly, D. tellermanica forms a clade with D. octaedra, but they are rather distantly related.

We can conclude that this work is a first step in the study of the basal branches of Lumbricidae. Representatives of the genus Dendrobaena from the Caucasus are particularly interesting in this respect, because they account for a large part of its species diversity.

**Fig. 4.**
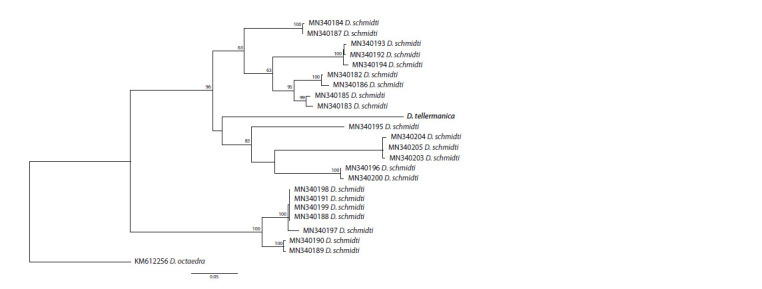
Phylogenetic tree based on the COX1 gene for the genus Dendrobaena using the Maximum Likelihood method.

Earlier we performed a genetic analysis of morphological forms of D. schmidti (Shekhovtsov et al., 2020b), demonstrating that it represents at least two separate species. On the phylogenetic tree constructed using the COX1 gene (Fig. 4), D. tellermanica was inside one of the branches of D. schmidti. We should note that single mitochondrial genes, including COX1, are unsuitable for phylogenetic reconstruction on the family level (Klarica et al., 2012; Shekhovtsov et al., 2016, 2020c), since they demonstrate poor resolution of the relationships between species and do not support the monophyly of most genera. COX1 is however of much use in the search for closely related species or genetic lineages. Moreover, there are thousands of COX1 sequences in the public databases and only a few mitochondrial genomes; e. g., mtDNA of D. schmidti has not been sequenced yet. Therefore, the tree on Fig. 4 is given only to demonstrate the close relationship of D. tellermanica and D. schmidti.

## Conclusion

The obtained preliminary results indicate that D. tellermanica could be treated as a subspecies of D. schmidti, as was believed earlier, or split D. schmidti into several species. The latte option is supported by the high genetic and morphological variation within this species. However, such conclusions would require an analysis based on several loci, including nuclear ones.

## Conflict of interest

The authors declare no conflict of interest.
